# The small GTPase RhoG regulates microtubule-mediated focal adhesion disassembly

**DOI:** 10.1038/s41598-019-41558-7

**Published:** 2019-03-26

**Authors:** Ashtyn Zinn, Silvia M. Goicoechea, Gabriel Kreider-Letterman, Debonil Maity, Sahezeel Awadia, Luis Cedeno-Rosario, Yun Chen, Rafael Garcia-Mata

**Affiliations:** 1 Department of Biological Sciences, University of Toledo, 2801 W. Bancroft St., MS601, BO3090, Toledo, OH 43606 USA; 2 Johns Hopkins University, Department of Mechanical Engineering, Latrobe Hall 223, 3400 North Charles St., Baltimore, MD 21218 USA

## Abstract

Focal adhesions (FA) are a complex network of proteins that allow the cell to form physical contacts with the extracellular matrix (ECM). FA assemble and disassemble in a dynamic process, orchestrated by a variety of cellular components. However, the underlying mechanisms that regulate adhesion turnover remain poorly understood. Here we show that RhoG, a Rho GTPase related to Rac, modulates FA dynamics. When RhoG expression is silenced, FA are more stable and live longer, resulting in an increase in the number and size of adhesions, which are also more mature and fibrillar-like. Silencing RhoG also increases the number and thickness of stress fibers, which are sensitive to blebbistatin, suggesting contractility is increased. The molecular mechanism by which RhoG regulates adhesion turnover is yet to be characterized, but our results demonstrate that RhoG plays a role in the regulation of microtubule-mediated FA disassembly.

## Introduction

Cell migration is a dynamic process involved in organogenesis, tissue maintenance, and cancer metastasis that depends on the ability of the cell to form physical contacts to the surrounding extracellular matrix (ECM)^1^. These contacts, known as focal adhesions (FA), are mechanosensitive structures that link the ECM to the actin cytoskeleton through integrin receptors^2^. The assembly and disassembly of FA drive cell migration through force transduction and the indirect regulation of actin polymerization and myosin II activity^3^. FA are formed at the leading edge, enabling the cell to adhere and stabilize protrusions, and must be disassembled behind the lamella or at the rear to allow the cell to detach, contract, and translocate forward^1^.

The composition of FA changes in response to internal and external mechanical tension, a process known as maturation^2,4^. FA formation begins at the front of the cell during initial protrusion^5^, and can be characterized by enriched areas of tyrosine-phosphorylation. These new adhesions, known as nascent adhesions, will continue to mature, growing in size, to become focal complexes which also contain vinculin^6^. Focal complexes stabilize the newly formed protrusions through their linkage to actin stress fibers (SF)^7^. Lastly, tensin-containing fibrillar adhesions aid the cell in ECM remodeling, providing a structural platform for migration^8^. The assembly and maturation of adhesions is a highly regulated process that has been well characterized. However, the molecular mechanisms that control FA disassembly are not well understood.

The Rho family of small GTPases plays a central role in the regulation of virtually every aspect of cell migration, including FA and stress fiber formation, lamellipodia dynamics, and actomyosin contractility^9^. Unfortunately, of the 20 members of the Rho GTPase family, most studies have focused on the role of the three best-characterized ones, RhoA, Rac1 and Cdc42. However, there are other lesser studied Rho GTPases, such as RhoG, which also play a role in cell migration^10–13^. Recently, we found that RhoG plays an important role in the regulation of invadopodia turnover^14^. Invadopodia are actin-rich adhesive structures used by cancer cells to degrade the ECM, and are built using many of the same components as FA^15^. This finding led us to believe that RhoG may regulate non-invasive cell migration as well, through FA dynamics. We found that RhoG regulates FA turnover, specifically the lifetime and maturation of FA. Our results also show that microtubule-mediated FA disassembly is involved in the regulation of FA turnover by RhoG.

## Results

### RhoG regulates focal adhesion formation and cell morphology

**Figure 1:**
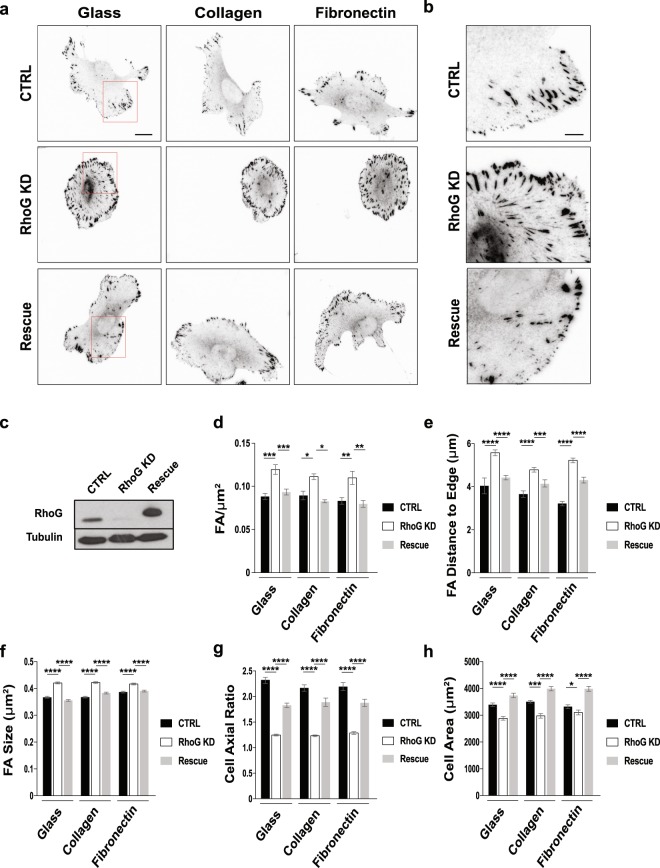
RhoG KD affects focal adhesion number and size, and cell morphology. RhoG expression was stably silenced in SUM159 cells using lentiviral shRNA (KD) and rescued transiently in RhoG KD cells using shRNA-resistant myc-RhoG wt (Rescue). Control cells stably express a non-targeting shRNA (CTRL). (**a**) Cells were plated on either non-coated glass coverslips or coverslips coated with collagen or fibronectin. Cells were then fixed and stained for focal adhesions using anti-vinculin antibodies. Boxes indicate areas enlarged in panel b. Scale bars: 20 μm. (**b**) Enlarged areas from highlighted regions in panel a. Scale bars: 1 μm. (**c**) Lysates from CTRL, RhoG KD and Rescue cells were immunoblotted with anti-RhoG antibodies. Tubulin was used as a loading control. (**d**–**h**) Focal adhesion properties were analyzed on all substrates using the Focal Adhesion Analysis Server (FAAS)^16^. (**d**) Number of adhesions per μm^2^. (**e**) Distance between the centroid of the focal adhesion and the edge of the cell. (**f**) Adhesions size (μm^2^). (**g**) Cell axial ratio. (**h**) Cell area (μm^2^). Results are shown as mean ± SEM (error bars). All data are results of 4 independent experiments where 20 cells per experiment were analyzed (n = 80). *p < 0.01, **p < 0.002, ***p < 0.001, ****p < 0.0001.

To characterize the role of RhoG in FA, we used a previously established SUM159 breast cancer cell line in which RhoG expression was stably silenced (RhoG KD), and control cells expressing a non-targeting shRNA (CTRL)^14^. We also rescued the expression of RhoG in RhoG KD cells using an adenovirus encoding a shRNA resistant myc-tagged wild-type RhoG (Rescue) (Fig. 1c)^14^. We then plated CTRL, RhoG KD and Rescue cells on uncoated glass coverslips, or coated with either collagen or fibronectin, and stained them for vinculin as a marker for FA. Our results showed that RhoG KD cells appeared to be slightly smaller and rounder in shape than control cells, and had more FA, in particular at the center of the cells (Fig. 1a,b). These results were reproducible regardless of the substrate tested. The cell shape/size and FA phenotypes were rescued by re-expressing myc-RhoG, indicating that the effects observed are due to the specific depletion of RhoG.

To confirm these observations, we quantified the number, size, and other FA characteristics using ImageJ and The Focal Adhesion Analysis Server (FAAS), a MatLab based algorithm designed to identify and quantify FA characteristics^16^. Quantification confirmed that when RhoG expression was silenced, there was a significant increase in the number of FA (Fig. 1d), as well as a significant difference in their localization, being found more frequently in the center of the cell in RhoG KD cells (Fig. 1e). We also found that FA are significantly larger in RhoG KD cells (Fig. 1f). Re-expression of RhoG restored the number, localization and size of FA to normal levels (Fig. 1d,e). A similar phenotype was observed in human MRC5 fibroblasts upon the knockdown of RhoG (Supplemental Fig. S1). We also measured the effect of silencing RhoG on cell morphology. We confirmed that RhoG KD cells were significantly rounder (Fig. 1g) and smaller (Fig. 1h). These changes in cell morphology may be directly related to the FA phenotype observed, which may compromise the ability of the cells to adhere and spread properly.

### RhoG silencing increases FA lifetime

**Figure 2:**
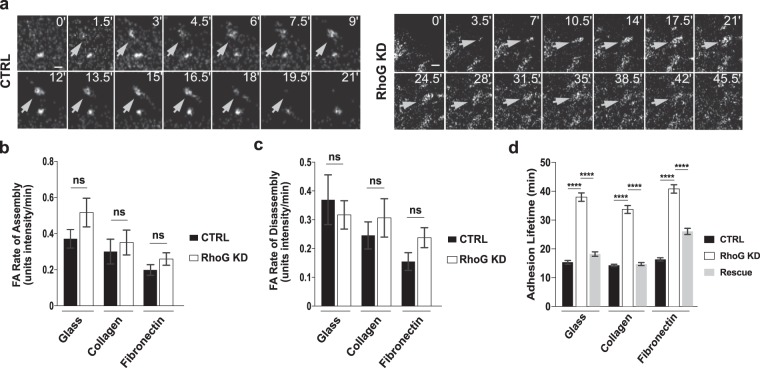
RhoG regulates adhesion lifetime, but not the rate of assembly and disassembly. CTRL and RhoG KD cells expressing GFP-paxillin were analyzed using time-lapse imaging every 10 seconds for approximately 40 minutes. (**a**) A representative adhesion tracked over the course of the indicated time (min), from appearance to disappearance, in CTRL (left panel) and RhoG KD (right panel) cells. Scale bars: 3 μm. (**b**,**c**) The rate of adhesion assembly/disassembly was calculated as described in the methods section. Data are results of at least 4 cells where at least 2 adhesions per cell were permitted into the data set, based on correlation criteria. n ≥ 17. (**d**) For adhesion lifetime, time-lapse images were acquired every 10 seconds for up to 1.5 hours. Data are results of at least 6 cells where at least 13 adhesions per cell were analyzed. n ≥ 104. All results are shown as mean ± SEM. ****p < 0.0001.

FA form at the leading edge and translocate inward towards the center of the cell as they mature. Most adhesions are rapidly turned over at the edge of the cell right after they form and never progress to mature FA. The increased number of FA in RhoG KD cells suggests that RhoG may be playing a role in the regulation of FA turnover. To explore this possibility, we measured FA assembly and disassembly rates in CTRL and RhoG KD cells using time-lapse microscopy in cell expressing GFP-paxillin (Supplemental Videos 1, 2). Figure 2a shows sample adhesions tracked in CTRL (left panel) and RhoG KD (right panel) cells. Our results showed that even though there was a trend indicating faster assembly rates in RhoG KD cells, there was no significant difference in the rate of assembly or disassembly of FA when RhoG was silenced (Fig. 2b,c).

Previous work has defined three characteristic phases during FA turnover: the assembly and disassembly phases, which are typically linear, and a stationary/mature phase in between, where the intensity of the adhesion remains relatively stable^16^. A prolonged stationary phase may help explain the increase in FA number observed in RhoG KD cells. If adhesions are more stable, FA would accumulate over time and translocate to the center of the cell as they mature. To test this hypothesis, we measured the lifetime of adhesions in extended movies (Supplemental Videos 3, 4) and found that adhesions lived significantly longer in RhoG KD cells (Fig. 2a,d). For example, in cells plated on glass, the average adhesion lifetime increased from 15.2+/−0.66 min in CTRL cells to 38.33+/−1.50 min in RhoG KD cells (Fig. 2d). We observed the same trend in cells plated on glass, collagen and fibronectin (Fig. 2d). Re-expression of RhoG rescued the FA lifetime to normal levels in all substrates. Our results show that the increased number of FA in RhoG KD cells may reflect an increase in their stability, as the FA live longer on average, and the assembly and disassembly rates are not significantly affected.

### RhoG KD promotes FA maturation

**Figure 3:**
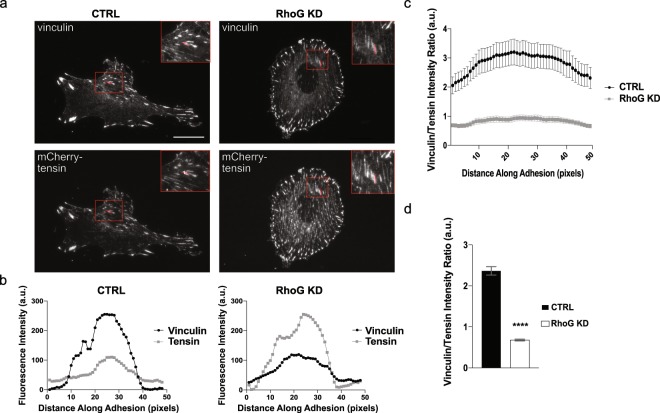
RhoG KD promotes focal adhesions maturation. (**a**) CTRL and RhoG KD cells expressing mCherry-Tensin (bottom panels) were fixed and stained with vinculin (top panels). Scale bars: 20 μm. (**b**) A line of 1-pixel width and 47 pixels length was drawn along the length of an adhesion and the intensity values along the line were plotted using ImageJ. Values for one representative adhesion, shown in zoomed insets in (**a**), are shown for both CTRL and RhoG KD cells. (**c**) Average ratio of vinculin/tensin intensity across an adhesion. (**d**) Average vinculin/tensin intensity ratio of a whole adhesion. (p < 0.0001). All results are shown as mean ± SEM. All data are results of 3 independent experiments where 5 cells and 10 adhesions per cell each were quantified. n = 150. ****p < 0.0001.

As adhesions mature, they not only change in size and location, but also in protein composition, resulting in distinct populations of adhesions within a single cell that can be identified using specific markers^6,17,18^. The most mature population of adhesions, fibrillar adhesions, are characteristically elongated streaks found at the center of the cell that contain the protein tensin. Based on their increased number and altered location, we predicted that the FA in RhoG KD cells were probably more mature when compared to CTRL FA. To test this prediction, we transiently transfected CTRL and RhoG KD cells with mCherry-tensin (Fig. 3a, bottom panels), processed them for immunofluorescence and stained them for vinculin (Fig. 3a, top panels). Our results showed that when RhoG was silenced, the signal from mCherry-tensin in FA appeared to be stronger when compared to vinculin. To quantify this difference, we first measured the intensity profiles of both vinculin and tensin along the distance of a single adhesion in CTRL (left panel) and RhoG KD (right panel) cells (Fig. 3a, inset). The results showed that in CTRL cells the intensity of vinculin along adhesions is higher than that of tensin (Fig. 3b, left panel). In contrast, the intensity of tensin was higher than that of vinculin in RhoG KD cells (Fig. 3b, right panel). Figure 3c confirms this result, showing the average vinculin/tensin ratio along multiple adhesions in different cells. To further confirm our findings, we calculated the average vinculin/tensin ratio using the intensity values from the whole adhesion, which also showed that, on average, the intensity of tensin was significantly higher than that of vinculin in RhoG KD FA (Fig. 3d).

To examine if RhoG KD also affects the composition of nascent adhesions, we co-stained CTRL, RhoG KD, and Rescue cells for vinculin and tyrosine-phosphorylated paxillin (Supplemental Fig. 2a). These proteins are both known to be recruited to adhesions found at the leading edge^19,20^. We saw no difference in the ratio of phospho-paxillin to vinculin along adhesions in RhoG KD cells compared to CTRL cells (Supplemental Fig. 2b).

Taken together, our results suggest that the increase in the lifetime of FA observed in RhoG KD cells, results in an increase in the number of mature FA.

### RhoG plays a role in lamellipodia dynamics

**Figure 4:**
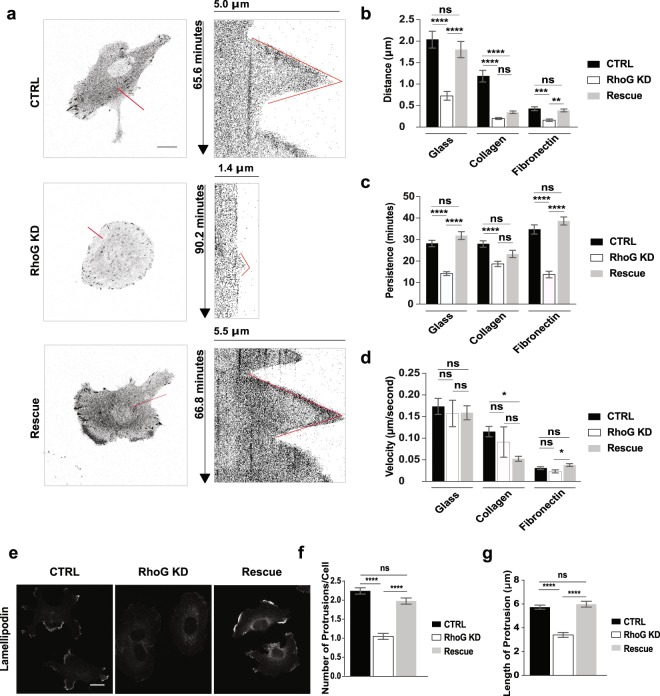
RhoG modulates protrusion dynamics and lamellipodia formation. CTRL, RhoG KD, and Rescue cells expressing GFP-paxillin were imaged every 10 seconds for up to 1.5 hours. (**a**) Representative kymographs constructed from red lines indicated on the left panel images for CTRL, RhoG KD, and Rescue cells. Scale bars: 10 μm. (**b**) Protrusion distance, (**c**) protrusion persistence, and (**d**) protrusion velocity were manually measured using ImageJ software. Protrusion data are results from 3 independent experiments where at least 2 cells were imaged and 3–6 kymographs per cell were generated. n ≥ 35. (e) CTRL, RhoG KD, and Rescue cells were stained for lamellipodia using anti-lamellipodin antibody. Scale bars: 10 μm. (**f**) The number of lamellipodia was manually measured using ImageJ software. Lamellipodia data are the results of 3 independent experiments where at least 32 cells were counted. n ≥ 135. (**g**) The length of protrusions was manually measured using ImageJ software. Data are the results of 3 independent experiments where at least 34 protrusions were measured. n ≥ 155. All results are shown as mean ± SEM. *p < 0.01, ***p < 0.001, ****p < 0.0001.

The regulation of FA assembly and turnover can influence the dynamics of protrusion formation at the leading edge of the cell^21^. Our time-lapse movies suggested that depletion of RhoG had a negative effect in frequency and amplitude of protrusion (Supplemental Videos 3, 4). Quantitative analysis of protrusion dynamics using kymography showed that the distance and persistence of protrusions were significantly decreased in RhoG KD cells (Fig. 4b,c). Both parameters were rescued to normal levels upon RhoG re-expression (Fig. 4b,c). In contrast, there was no difference in the velocity of protrusion formation between CTRL, RhoG KD and Rescue cells (Fig. 4d). These results were consistent across all substrates tested. The exception was collagen, where both distance and persistence were increased slightly but were not significantly rescued by RhoG re-expression (Fig. 4b,c). Supporting these results, RhoG KD cells stained for lamellipodia, using an antibody against lamellipodin^22^, showed a significant decrease in the number and length of protrusions compared to CTRL and Rescue cells (Fig. 4e–g). Our data suggest that RhoG’s regulation of FA may be affecting the ability of the cell to form and maintain stable lamellipodia.

### RhoG regulates the alignment of focal adhesions and stress fibers

**Figure 5:**
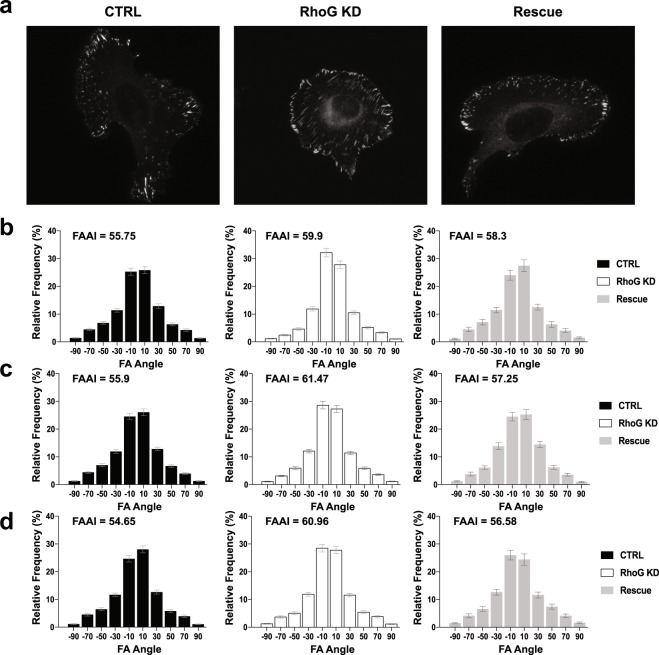
RhoG KD increases focal adhesions alignment. (**a**) Representative pictures of adhesion alignment in CTRL, RhoG KD, and Rescue cells plated on glass and stained for focal adhesions using anti-vinculin antibody. Scale bars: 10 μm. (**b**–**d**) Adhesion alignment was measured using the FAAS^16^, and then graphed as a frequency distribution. An index, termed the focal adhesion alignment index (FAAI) was calculated, as previously described by Wu *et al*.^23^, to measure the overall alignment of adhesions within individual cells (inset on all graphs). (**b**) The FAAI of CTRL, RhoG KD and Rescue cells plated on glass. (**c**) The FAAI of CTRL, RhoG KD and Rescue cells plated on fibronectin. (**d**) The FAAI of CTRL, RhoG KD and Rescue cells plated on collagen. All results are shown as mean ± SEM. All data are results of 3 independent experiments where 20 cells per experiment were analyzed. n = 60.

Silencing RhoG not only affected the composition and dynamics of individual adhesion, but also the pattern of the entire adhesion population within the cell. We noticed that in most RhoG KD cells, FA were aligned along an axis within individual cells (Fig. 5a). This axis was not the same for all cells, as individual cells developed their own pattern of alignment regardless of neighboring cells. To confirm that RhoG KD does affect adhesion alignment, we quantified global FA alignment, using a previously developed parameter, the focal adhesion alignment index (FAAI), which measures the deviation of adhesion angles from the most frequent or dominant angle observed in the whole cell^23^. The higher the FAAI, the greater is the global adhesion alignment within a single cell. We found that adhesion alignment was significantly increased in RhoG KD cells in all substrates tested (Fig. 5b–d). The increase in FA alignment was rescued by re-expressing RhoG in KD cells (Fig. 5b–d), except for Rescue cells on glass, where FAAI decreased slightly, but the difference was not significant from KD cells. This may be due to the axial ratio, or length, of adhesions in Rescue cells plated on glass. Since the FAAI requires that an adhesion’s axial ratio be large enough to determine directionality, if adhesions are more rounded, then the sample may be biased towards larger adhesions that are more aligned. Without the presence of substrate, we found that adhesions in Rescue cells are significantly smaller (Fig. 1f), supporting this notion.

**Figure 6:**
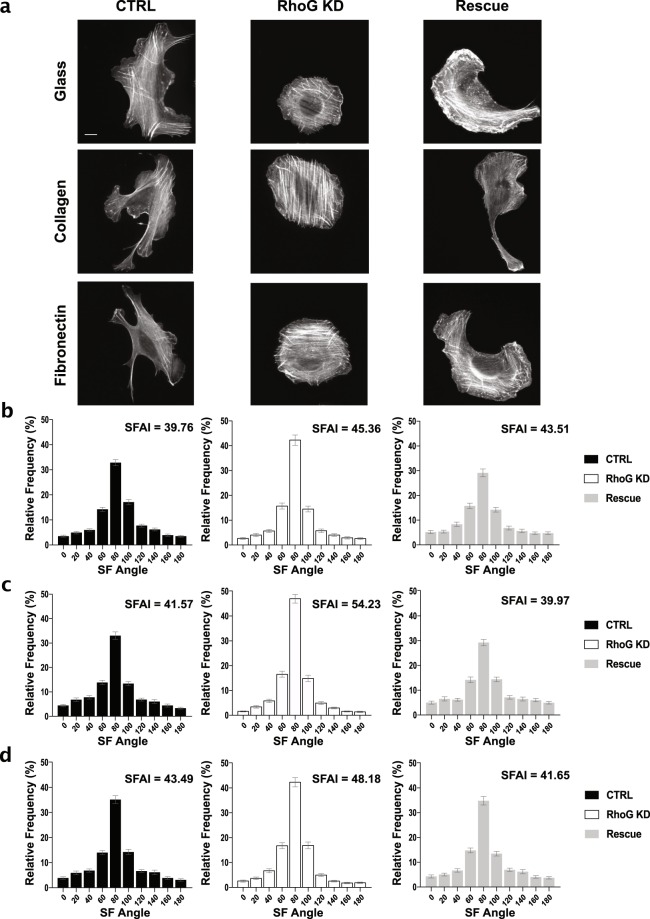
Silencing RhoG increases stress fibers alignment. (**a**) CTRL, RhoG KD, and Rescue cells were plated overnight on non-coated glass coverslips, or on coverslips coated with collagen or fibronectin. Cells were then fixed and stained for F-actin using Alexa Fluor phalloidin. Scale bars: 10 μm. (**b**–**d**) Actin stress fiber alignment was measured using CurveAlign (V3.0 Beta 2) software. Angles from individual cells were graphed as a frequency distribution and then rotated so that 90 degrees was the dominant bin. SF alignment distributions of CTRL, RhoG KD, and Rescue cells on glass (**a**), collagen (**b**), and fibronectin (**c**) with the value for the stress fiber alignment index (SFAI) displayed at inset in each graph. All results are shown as mean ± SEM. All data are results of 3 independent experiments where 20 cells per experiment were analyzed. n = 60.

Since most SF are attached at one or both ends to FA, we also evaluated the alignment of SF. We stained CTRL, RhoG KD, and Rescue cells for F-actin (Fig. 6a), and quantified actin alignment using CurveAlign, a software that can identify fibers and measure their alignment^24^ (Fig. 6b–d). We also adapted the FAAI approach to calculate a SF alignment index (SFAI) (Fig. 6b–d, inset on graphs). Our data showed an increase in SF alignment in RhoG KD cells, an effect that could be reversed by re-expression of RhoG (Fig. 6b–d). These results were observed on collagen and fibronectin as well. Together, these data suggest that RhoG-mediated regulation of FA dynamics also affects the global coordination of FA and the actin cytoskeleton within a cell.

### RhoG regulates actomyosin contractility

**Figure 7:**
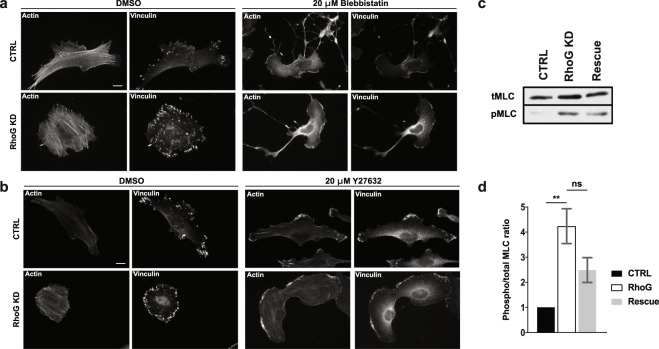
RhoG KD cells are responsive to inhibitors of contractility. (**a**) CTRL (top panels) and RhoG KD (bottom panels) cells were plated on glass coverslips overnight and then treated with DMSO or 20 μM blebbistatin for 10 min. Cells were then fixed and stained for vinculin and F-actin. Scale bars: 10 μm. (**b**) CTRL (top panels) and RhoG KD (bottom panels) cells were plated on glass coverslips overnight and then treated with DMSO or 100 μM Y-27632 for 10 min. Cells were then fixed and stained for vinculin and F-actin. Scale bars: 10 μm. (**c**) CTRL, RhoG KD, and Rescue cells were blotted for total and phospho-MLC and quantified by densitometry (**d**). Data are the results of three independent experiments. n = 3. All results are shown as mean ± SEM., **p < 0.01.

FA serve a mechanical role by forming a tether between the cytoskeleton and the ECM, thus acting as anchor sites for the cell to exert force onto the substrate^25^. The recruitment of mechanosensitive proteins, resulting in formation and growth of FA, and alignment of FA can be induced through the application of external force or activation of proteins involved in contractility pathways, such as RhoA^26^. Additionally, contractility plays a role in the polymerization and stabilization of actin filaments through activation of RhoA and the inhibition of cofilin, respectively^26,27^. We observed a phenotype with an increased number of FA and alignment of both FA and SF, which strongly suggested that cells were more contractile in the absence of RhoG. This was confirmed by treating CTRL and RhoG KD cells with contractility inhibitors. Both cell types displayed sensitivity to blebbistatin, a myosin II inhibitor, and Y27632, which inhibits ROCK, as indicated by the disassembly of SF and FA, suggesting that the SF are functional and contractile (Fig. 7a,b).

An increase in contractility is usually associated with high levels of phosphorylated myosin regulatory light chain (MLC)^28^. The levels of phospho-MLC are regulated by the balance between the activity of myosin light chain kinase (MLCK) and MLC-phosphatase^29,30^. Based on our results, we expected to see an increase in MLC phosphorylation in the absence of RhoG. This was confirmed by measuring the ratio of phospho-MLC to total-MLC by western blot in CTRL, RhoG KD and Rescue cells (Fig. 7c,d). It is worth noting that we also observed an increase in the amount of total-MLC in RhoG KD cells (Fig. 7c,d), which shows that the expression levels of MLC are also regulated in response to changes in RhoG.

### RhoG KD affects microtubule dynamics

**Figure 8:**
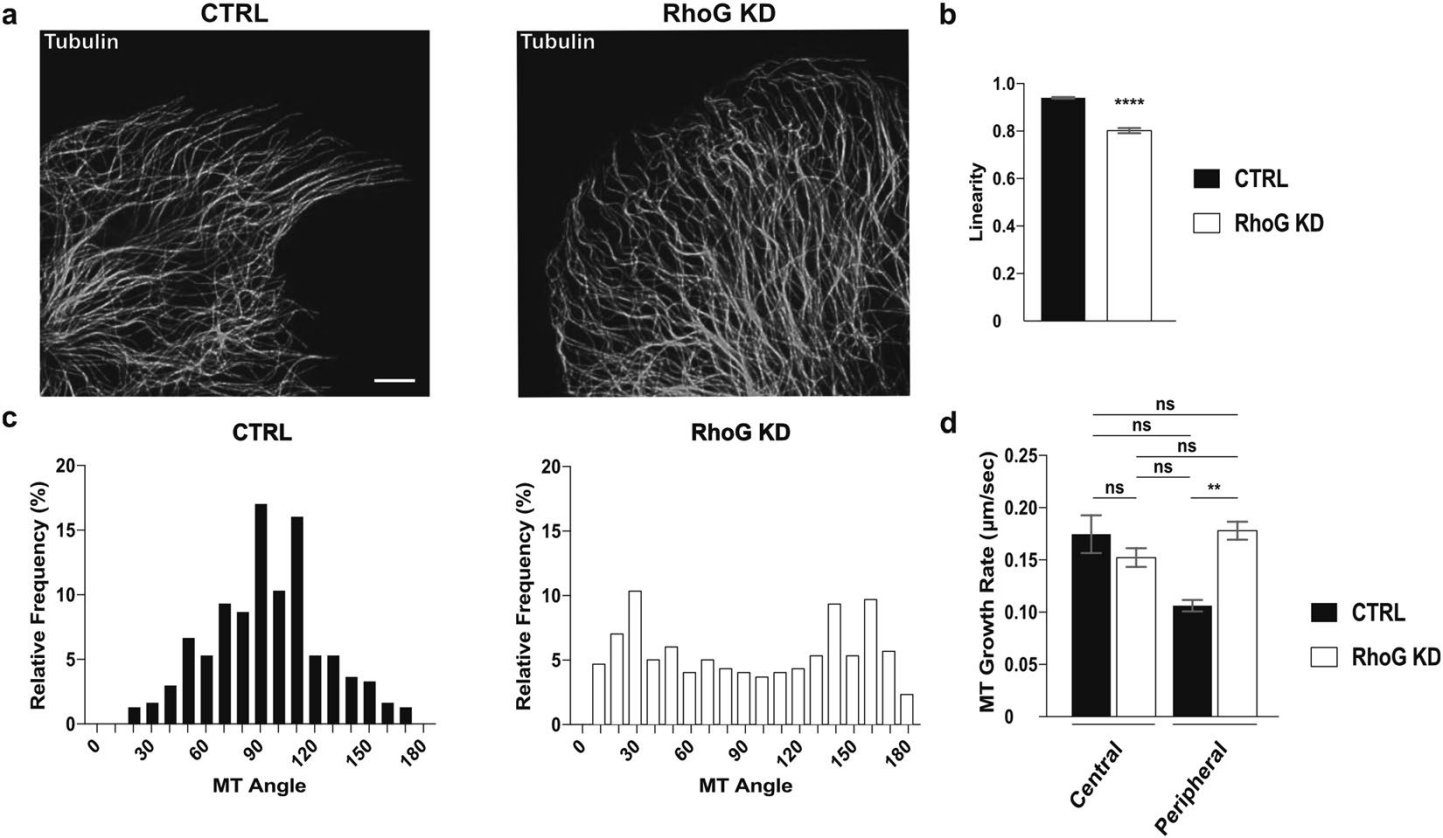
RhoG regulates MT outgrowth. (**a**) CTRL and RhoG KD cells were plated overnight on non-coated, glass coverslips. Cells were then fixed and stained for MT using anti-tubulin antibodies. Scale bars: 5 μm. (**b**) Linearity of MT in CTRL and RhoG KD cells, measured as the actual distance divided by the shortest distance between 2 points. Data are results from 3 independent experiments where 5 cells and 15 MT per cell were measured. n = 200. (**c**) The relative frequency of the angle of the MT relative to the cell edge in CTRL (left panel) and RhoG KD (right panel) cells. Results are from 3 independent experiments where 5 cells and 20 MT per cell were measured. n = 300. (**d**) CTRL and RhoG KD cells were transfected with EB3-mRFP. Cells were imaged every 3 seconds for approximately 8.5 minutes. Central or peripheral MT growth rate was manually measured for 5 MT in 12 cells using the MTrackJ plugin for ImageJ software. n = 60. All results are shown as mean ± SEM. ****p < 0.0001.

The microtubule (MT) cytoskeleton has also been shown to play a role in the regulation of FA turnover^31^. MT can target adhesions located at the edge of the cell to promote their disassembly^32–34^. A defect in the capturing process could prolong adhesion life, leading to larger populations of mature adhesions, like those seen in RhoG KD cells. To test this hypothesis, we first stained CTRL and RhoG KD cells for α-tubulin to examine their MT distribution (Fig. 8a). Our results revealed a distinct difference in the MT pattern between CTRL and RhoG KD cells. In CTRL cells, MT irradiated from the center of the cell to the periphery, where each individual MT typically reached the edge of the cell at an angle perpendicular to the cell membrane. In contrast, MTs in RhoG KD cells curved when they reached the cell periphery and adopted an angle that was often parallel to the cell edge (Fig. 8a). Quantification of MT linearity showed a significant decrease in RhoG KD cells, confirming that the MT are more curved in the absence of RhoG (Fig. 8b). In addition, the MT angle relative to the cell’s edge shows a larger percentage of CTRL MT that are perpendicular to the cell edge, whereas in KD cells there is a higher frequency of MTs that align parallel to the cell membrane (Fig. 8c).

The angle of the MT relative to the cell edge can be indicative of their growth rate. In normal cells MT tend to grow faster at the center of the cell and slow down at the edge due to the combined action of several factors, including targeting to FA^35,36^. In cells with abnormal MT targeting to FA, the MTs at the edge of the cell do not slow down, causing them to continue growing, eventually curving parallel to the edge of the cell^36^. We used live imaging to analyze the dynamics of the MT plus-end marker EB3-mRFP cells to examine MT growth rate in CTRL vs. RhoG KD cells (Supplemental Videos 5, 6). We found that there was no difference in the MT growth rate measured at the cell center. However, at the cell periphery, MT grew significantly faster in RhoG KD cells when compared to CTRL (Fig. 8d). These data suggest that RhoG regulates MT dynamics at the leading edge, which may impact MT-mediated FA disassembly. Thus, the absence of RhoG would lead to increased FA lifetimes, which would result in an increase in FA numbers that progress to mature FA at the center of the cell.

### RhoG KD inhibits MT-mediated FA disassembly

As mentioned above, targeting of FAs by growing MTs coincides with their disassembly^32–34^. When cells are treated with nocodazole, the depolymerization of MTs induces the stabilization of FA^37,38^. Nocodazole washout stimulates the regrowth of MT after depolymerization, which induces a rapid disassembly of FA^34^. Given that RhoG KD affected both FA turnover and MT dynamics, we considered that RhoG might be playing a role in the regulation of MT-dependent FA disassembly. To test this possibility, we performed nocodazole washout experiments in CTRL and RhoG KD cells.

**Figure 9:**
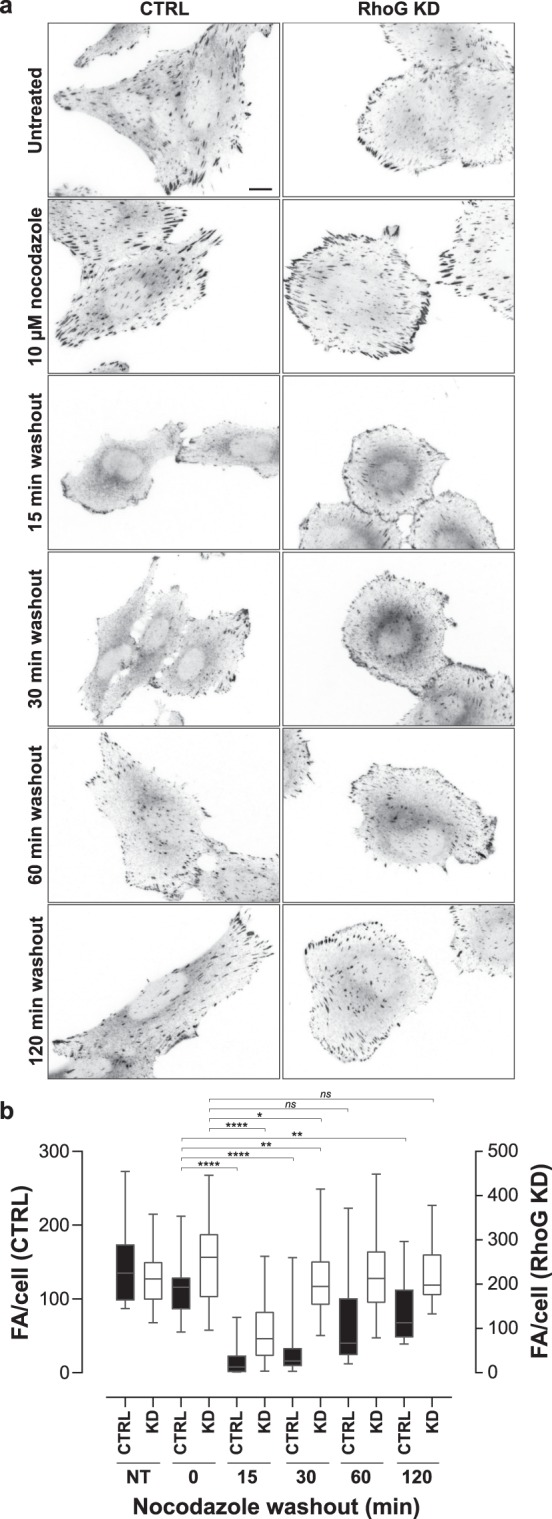
RhoG KD inhibits MT-mediated FA disassembly. (**a**) CTRL and RhoG KD cells were starved overnight and then treated with nocodazole at 10 μM for 1 h. Following treatment cells were washed once with SFM and then incubated with SFM for the indicated times (nocodazole washout). After washout the cells were fixed and stained for vinculin. Scale bar: 10 μm. (**b**) The number of adhesions was calculated manually using Image J. Data are the results of 2 independent experiments where at least 25 cells were analyzed per condition. All results are shown as mean ± SEM. *p < 0.05, **p < 0.01, ****p < 0.0001.

Our results showed, as expected, that FA size increased in both CTRL and RhoG KD cells treated with nocodazole (Fig. 9a). In CTRL cells, nocodazole washout promoted a rapid decrease in the number of FA. The maximum decrease in FA number was observed at 15 minutes in CTRL cells (Fig. 9a,b). FA number started to increase at 30 minutes and continued to increase steadily through the duration of the washout treatment. Importantly, after 120 min of washout the number of FA in CTRL cells was still significantly lower than in non-treated or nocodazole treated CTRL cells. In contrast, in RhoG KD cells FAs remained stable through the duration of the nocodazole washout. The decrease in FA number was significantly less pronounced than in CTRL cells, and the recovery was also faster, reaching non-treated or nocodazole treated levels after 30 min of washout (Fig. 9a,b). Quantification showed that in CTRL cells the number of FA decreased approximately 8-fold, whereas in RhoG KD cells the reduction was only 2.3-fold. MT staining showed that MT regrowth was not affected when RhoG was silenced (Supp. Fig. 3), suggesting that RhoG is required specifically for the process of FA disassembly after MT regrowth.

## Discussion

RhoG has been previously shown to play a role during cell migration, but the mechanism has not been further explored^10–13^. Here we show that RhoG regulates the number, size, lifetime and maturation stage of FA, as well as SF contractility, suggesting that the role of RhoG in cell migration may be mediated through the regulation of FA turnover. Our results also demonstrate that RhoG controls FA turnover through the regulation of MT-mediated FA disassembly.

FA form at the leading edge and translocate towards the center of the cell as the cell body moves forward. Eventually, FA disassemble either behind the lamella or at the rear of the cell allowing the cell to move forward. As FA are disassembled, their components are recycled to new adhesions at the leading edge, completing the cycle^1,39^. Thus, the assembly and disassembly of FA must be precisely controlled for cells to migrate properly. Initially, we predicted that the adhesion phenotype in RhoG KD cells was the result of an increased rate of assembly and/or decreased disassembly, resulting in a net accumulation of FA at the center of the cell. However, we found no change in the assembly or disassembly rates of FA, but rather an increase in their lifetime.

The maturity of adhesions can be characterized by the diversity in protein composition at the different stages, which allows FA to carry out various functions that aid cell migration^2,39,40^. Due to the increase in number of FA and their location in RhoG KD cells, we hypothesized that RhoG was regulating maturation. We found that there was an increase in the amount of tensin in FA when RhoG was silenced, indicating more mature adhesions. Taken together, our results show that in the absence of RhoG, FA become more stable and live longer, leading to an increased number of mature FA found at the center of the cell. We have also shown that RhoG depletion impairs leading edge protrusion. The slower rate of FA turnover combined with altered lamellipodia dynamics are the most likely causes for observed defects in cell migration^10–13^.

Contractile forces have long been the focus of studies elucidating the regulation of adhesion formation and the ability of the cell to respond to the environment^25^. Both MT and actin SF coordinate the regulation of contractility^41,42^. This dynamic interplay between cytoskeletal elements plays an important role in the balance between assembly and disassembly of FA^3^. We observed a phenotype where FA and SF were aligned along a common axis in individual RhoG KD cells. This was a particularly striking observation because events of comparable FA and SF alignment were observed where mechanical force was applied externally to the cell^43–47^. The phenotype adopted by RhoG KD cells, i.e. round cells, with more FA and thicker SF, suggested an increase in contractility. This was confirmed by their sensitivity to contractility inhibitors and increased levels of pMLC. Contractility of SF is especially important in cell migration because mechanical force held in the SF is exerted upon the ECM through FA, allowing the cell to progress forward.

The molecular mechanisms by which RhoG regulates these processes are unclear. However, our results suggest that MT-mediated FA disassembly may be playing a role in the regulation of FA lifetime by RhoG. MT target FA at the cell edge and induce their disassembly, in a process known as capturing^32–34^. This is accomplished through a recently identified complex of proteins that aids in MT capture near FA^48^. When MT do not efficiently capture at FA, their growth rates and angle relative to the cell edge are affected^35,36^. Here we show that the speed and directionality of MT outgrowth are affected in the absence of RhoG, which may be indicative of impaired capture and reduced number of disassembly events. This in turn would translate into increased number of FA over time, which would eventually mature and accumulate at the center of the cell. Nocodazole washout experiments also show that MT-dependent disassembly is impaired in the absence of RhoG, preventing or slowing down the disassembly of FA over time upon washout. We plan to further explore the possibility of RhoG’s role in MT capture at FA, contributing to cell migration defects.

It is not clear whether RhoG localizes to FA and we have not been able to stain for endogenous RhoG with the currently available antibodies. Exogenously expressed RhoG localizes primarily to the cytosol, and at the perinuclear region where it targets to the Golgi through its association with RhoGDI3^49–51^. Local concentrations of RhoG have also been detected at the cell periphery, at places that may overlap with FA^13,49^. Interestingly, a proteomics study identified RhoG as one of the proteins that was reproducibly recovered in isolated FA, suggesting that a fraction of RhoG may be targeted to FA^52^.

Our results suggest that a RhoG-specific GEF activates RhoG at the time of disassembly. We have previously identified SGEF and Trio as upstream regulators of RhoG signaling during invadopodia and circular dorsal ruffles disassembly^14,53^. However, our preliminary results using shRNAs targeting known RhoG-specific, including SGEF, Trio, Ephexin 4, PLEKHG6, Vav1-3, were inconclusive and suggests that none of them are involved in regulating RhoG in FA. This could be attributed to an incomplete KD, or to compensation by another GEF in response to the KD. Alternatively, RhoG may be regulated at FA by another yet to be characterized RhoG GEF (most Rho-GEFs have not been tested for RhoG specificity). We are continuing our efforts to identify the RhoG-GEF in FA.

In conclusion, we have identified a novel role for RhoG in cell migration, regulating the disassembly of FA in a process that involves MT. The molecular mechanism by which RhoG controls these processes is not known. The regulation of Rho GTPase activity relies on a complex system where multiple upstream GEFs and GAPs can regulate a single Rho GTPase, which can then activate a vast array of downstream effectors. The identification of the upstream regulators and the downstream effectors will be key to fully understand the role of RhoG during cell migration.

## Materials and Methods

### Reagents and antibodies

The following antibodies were used: mouse anti-RhoG (Santa Cruz, sc-1007), mouse anti-myc (9E10) (Santa Cruz, sc-40), mouse anti-vinculin (mouse) (Sigma, V9131), rabbit anti-vinculin (Thermo-Fisher, 700062); rabbit anti-phospho-paxillin (Y118) (Cell Signaling, 2541), rabbit anti-lamellipodin (Cell Signaling, 91138), rabbit anti-Myosin Light Chain and rabbit anti-phospho-Myosin Light Chain (Ser 19) (Cell Signaling, 3671, 3672) mouse anti-alpha tubulin (Sigma, T9026), rabbit anti-tubulin (Abcam, ab18207); Alexa Fluor-488 and Alexa Fluor-594 anti-mouse IgG and anti-rabbit IgG conjugated secondary antibodies and Alexa Fluor-488 and Alexa Fluor-594 Phalloidin (Life Technologies). HRP-conjugated anti-mouse, anti-rabbit and anti-goat secondary antibodies (Jackson Immunoresearch). Hoechst 33342 (AnaSpec Inc., 83218).

Fibronectin (a gift from Keith Burridge, UNC-Chapel Hill, Chapel Hill, NC) and collagen type I (Thermo Fisher, A1048301) were used at indicated concentrations to coat coverslips. The contractility inhibitors (−)-blebbistatin (EMD Millipore) and Y27632 (LC Laboratories) were used as indicated. Nocodazole (Sigma) was used as indicated below.

### cDNA constructs

mCherry-Tensin-C14 (a gift from Michael Davidson, Addgene plasmid #55143). mCherry-LifeAct (a gift from Jaap van Buul, Sanquin Institute, Amsterdam, Netherlands). GFP-EB3 (a gift from Kristen Verhey, University of Michigan, Ann Arbor, MI). Paxillin-GFP (a gift from Channing Der, UNC-Chapel Hill, Chapel Hill, NC) was subcloned into pAd/CMV/V5-DEST using Gateway recombination technology (Life Technologies). Virus particles were produced using the Virapower Adenoviral Expression System (Life Technologies). The shRNA resistant mycRhoG construct used for rescue experiments has been previously described^14^.

### Lentiviral shRNA constructs and transduction

pLKO.1 lentiviral non-targeting shRNA control was from Sigma (SHC0161EA). pLKO.1 shRNA for human RhoG (#5 TRCN0000048022) were from Open Biosystems (Huntsville, AL). Lentiviruses were prepared at the Lenti-shRNA Core Facility, University of North Carolina (Chapel Hill, NC). Cells were infected with lentivirus particles overnight. The following day, the infection media was removed and replaced with complete medium containing puromycin (2.5 µg/ml) (Sigma) to select for shRNA expressing cells and total cell lysates were subjected to Western blot analysis for protein expression as described. For some shRNAs, single cell colonies were isolated by serial dilution.

### Cell Lines

Human SUM159 cells were a gift from Dr. Carol Otey (UNC-Chapel Hill, Chapel Hill, NC) and were cultured in Ham’s F12 media (Corning) supplemented with 10% fetal bovine serum (FBS, Rocky Mountain Biologicals), 0.5 μg/ml hydrocortisone (Sigma), and 2.5 μg/ml insulin (Life Technologies). Human MRC5 fibroblasts were purchased from ATCC and cultured in Dulbecco’s modified Eagle’s medium (DMEM, Corning) supplemented with 10% FBS. All cells were grown at 37 °C and 5% CO_2_. All experiments were conducted with early passage cells that were passaged no more than 15 times. Mycoplasma was tested regularly by staining with Hoechst 33342 (Anaspec).

### Imaging and analysis

When indicated, acid washed coverslips were coated with 25 µg/ml (fixed imaging) or 50 µg/ml (live-imaging) fibronectin for 24 hours at 4 °C. Rat tail type I collagen coated coverslips were purchased from Neuvitro (Cat. GG-12-1.5-Collagen). Prior to plating cells, collagen and fibronectin coated coverslips were blocked with 1.5% BSA in PBS for 1.5 hours at 37 °C.

Immunofluorescence assays were performed as described previously^54^. Briefly, cells were fixed for 10 min with 3.7% paraformaldehyde, and quenched with 10 mM ammonium chloride (for experiments involving fixed-imaging of MT, cells were fixed with −20 °C methanol or glutaraldehyde). Cells were then permeabilized with 0.1% Triton X-100 in PBS for 10 min. The coverslips were then washed with PBS and blocked in PBS, 2.5% goat serum (Sigma), 0.2% Tween 20 for 5 min followed by 5 min blocking in PBS, 0.4% fish skin gelatin (Sigma), and 0.2% Tween 20. Cells were incubated with primary antibody for 1 h at room temperature. Coverslips were then washed with PBS, 0.2% Tween 20 and incubated with Alexa Fluor 488 or 594 secondary antibodies for 45 min, washed as described above and mounted on glass slides in MOWIOL mounting solution. Images were acquired on an Olympus IX81 inverted microscope using a PlanApo N 60×/1.42 oil objective lens and a XM10 camera (Olympus).

Live imaging was performed for the indicated times in a Leica SP8 confocal microscopy using a PlApo CS2 N 63×/1.4 objective (Leica), equipped with an environmental chamber that controls temperature, CO2, and humidity (Tokai Hit). Images were processed using ImageJ software.

Images were threshold adjusted and then converted to binary prior to manually tracing edges for cell area measurements and perimeter coordinates, or for measuring the long axis and short axis for axial ratio. Focal adhesions characteristics were quantified using the Focal Adhesion Analysis Server (FAAS) (http://faas.bme.unc.edu)^16^.

Intensity profiles were measured by manually drawing a 1-pixel width line along the long axis of an adhesion using ImageJ software. Mean intensity value was measured at each pixel along the line. Adhesion perimeters were manually drawn in order to measure the total intensity of an adhesion.

### Measuring Adhesion Dynamics

Cells were infected with adenovirus encoding GFP-paxillin for 24 hours. They were then plated in a glass-bottom MatTek plate (MatTek corporation) for an additional 24 hours. Images were acquired every 10 seconds for the indicated duration.

Adhesion assembly and disassembly was measured using a previously described method^55^. Briefly, adhesions were chosen manually using image stacks with an applied grid in ImageJ. Mean intensity value of adhesions were measured manually in ImageJ. Assembly was defined as the time between appearance to peak max intensity, and disassembly the time between peak max intensity to disappearance. An adhesion had to fully appear and disappear to be included in data. Background was subtracted using a randomly selected area from within the cell, absent of adhesion, at each frame. Max intensity values were graphed, and linear regression values and slope of the line were calculated in Microsoft Excel. In order for an adhesion to be included in the data set, a minimum linear regression value of 0.6 was required. The slope of the line represents the rate of assembly or disassembly. Adhesion lifetime was tracked manually, defined as the total time between appearance and disappearance.

### Measuring Protrusion Dynamics

Images acquired for adhesion lifetime measurements were used to measure protrusion dynamics. Velocity, persistence, and distance were measured manually using ImageJ software, as previously described^56^. Briefly, a line of 1-pixel width was drawn perpendicular to a protrusion and a kymograph was generated using the KymographBuilder plugin for ImageJ. Measurements were then manually measured using ImageJ.

### Measuring MT Dynamics

Cells were transiently transfected with mCherry-EB3 for 24 hours. They were then plated in a glass-bottom Matek 35 mm plate for an additional 24 hours. Images were acquired every 2.95 seconds. MT velocity was manually measured using the ImageJ MTrackJ plugin. MT linearity, defined as the shortest distance between 2 points divided by the actual distance, and angle relative to the cell edge were manually measured using ImageJ software.

### Nocodazole washout experiment

Nocodazole washout experiments were performed as previously described by Ezratty and colleagues^34^. Briefly, CTRL and RhoG KD SUM159 cells were starved overnight in serum-free media (SFM). The cells were then treated with nocodazole at 10 μM in SFM for 1 h. Following treatment cells were washed once with SFM and the incubated with SFM for the indicated times (nocodazole washout). After washout the cells were fixed and processed for immunofluorescence as described in Methods.

### Statistical analysis

Statistical analysis was performed using GraphPad Prism 7. One-way ANOVA was used to compare multiple condition assays, and unpaired t-test to compare independent groups.

## Supplementary information

Supplementary Figures

Supplementary video 1

Supplementary video 2

Supplementary video 3

Supplementary video 4

Supplementary video 5

Supplementary video 6

## Data Availability

No datasets were generated or analysed during the current study.
