# Supplementary material for: The small GTPase RhoG regulates microtubule-mediated focal adhesion disassembly
Source: Sci Rep. 2019 Mar 26;9:5163. doi: 10.1038/s41598-019-41558-7 (PMC6435757; doi:10.1038/s41598-019-41558-7)
Supplement: Supplementary file 1 — Supplementary Figures [file 41598_2019_41558_MOESM1_ESM.pdf]

Supplementary Material

The small GTPase RhoG regulates microtubule-mediated focal adhesion disassembly

Ashtyn Zinn[†], Silvia M. Goicoechea[†], Gabriel Kreider-Letterman[†], Debonil Maity[¶], Sahezeel Awadia[†], Luis Cedeno-Rosario[†], Yun Chen[¶], and Rafael Garcia-Mata[†]

Affiliations:

† Department of Biological Sciences, University of Toledo. 2801 W. Bancroft St., MS601, BO3090, Toledo, OH 43606. TE: 419-530-1996. E-mail: rafael.garciamata@utoledo.edu

¶ Johns Hopkins University, Department of Mechanical Engineering. Latrobe Hall 223, 3400 North Charles St., Baltimore, MD 21218.

Supplemental figure 1. RhoG regulates FA in MRC5 fibroblasts. (a) RhoG expression was stably silenced in MRC5 fibroblasts using lentiviral encoding shRNA (RhoG KD). CTRL cells express a non-targeting shRNA. (b) CTRL and RhoG KD cells were plated on either non-coated coverslips, or coated with collagen or fibronectin, and stained for FA using anti-vinculin antibodies. Scale bar: 20 μm.

Supplemental figure 2. RhoG regulates vinculin and phospho-paxillin colocalization. (a) CTRL, RhoG KD, and Rescue cells were stained for vinculin (top panels) and phospho-paxillin (bottom panels). Scale bars represent 3 μm distance. (b) A line of 1-pixel width and 40 pixels length was drawn across an adhesion and intensity values were plotted using ImageJ. Values for one representative adhesion, marked as a ROI in images, are shown for CTRL, RhoG KD, and Rescue cells. All results are shown as mean ± SEM. All data are results of 3 independent experiments where 5 cells and 10 adhesions per cell each were quantified. n = 150. $^*p<.02$.

Supplemental figure 3. RhoG KD does not affect MT-regrowth after nocodazole washout. CTRL and RhoG KD cells were starved overnight and then treated with nocodazole at 10 μM for 1 h. Following treatment cells were washed once with SFM and the incubated with SFM for the indicated times (nocodazole washout). After washout the cells were fixed and stained for tubulin. Scale bar: 10 μm.

Supplemental video 1. FA dynamics in CTRL cells. CTRL cells were transiently transfected with GFP-paxillin and imaged every 10 seconds for approximately 40 minutes using confocal microscopy. Videos play at 16 frames per second. Time stamps display min:sec.

Supplemental video 2. FA dynamics in RhoG KD cells. RhoG KD cells were transiently transfected with GFP-paxillin and imaged every 10 seconds for approximately 40 minutes using confocal microscopy. Videos play at 16 frames per second. Time stamps display min:sec.

Supplemental video 3. Lamellipodia protrusions and FA lifetime in CTRL cells. CTRL cells were transiently transfected with GFP-paxillin and imaged every 10 seconds for approximately 1 hour using confocal microscopy. Videos play at 16 frames per second. Time stamps display min:sec.

Supplemental video 4. Lamellipodia protrusions and FA lifetime in RhoG KD cells. CTRL cells were transiently transfected with GFP-paxillin and imaged every 10 seconds for approximately 1 hour using confocal microscopy. Vidoes play at 16 frames per second. Time stamps display min:sec.

Supplemental video 5. Microtubule dynamics in CTRL cells. CTRL cells were transiently transfected with EB3-mRFP and imaged every 2.5 seconds for approximately 8.5 minutes using confocal microscopy. Time stamps display min:sec.

Supplemental video 6. Microtubule dynamics in RhoG KD cells. CTRL cells were transiently transfected with EB3-mRFP and imaged every 2.5 seconds for approximately 8.5 minutes using confocal microscopy. Time stamps display min:sec.

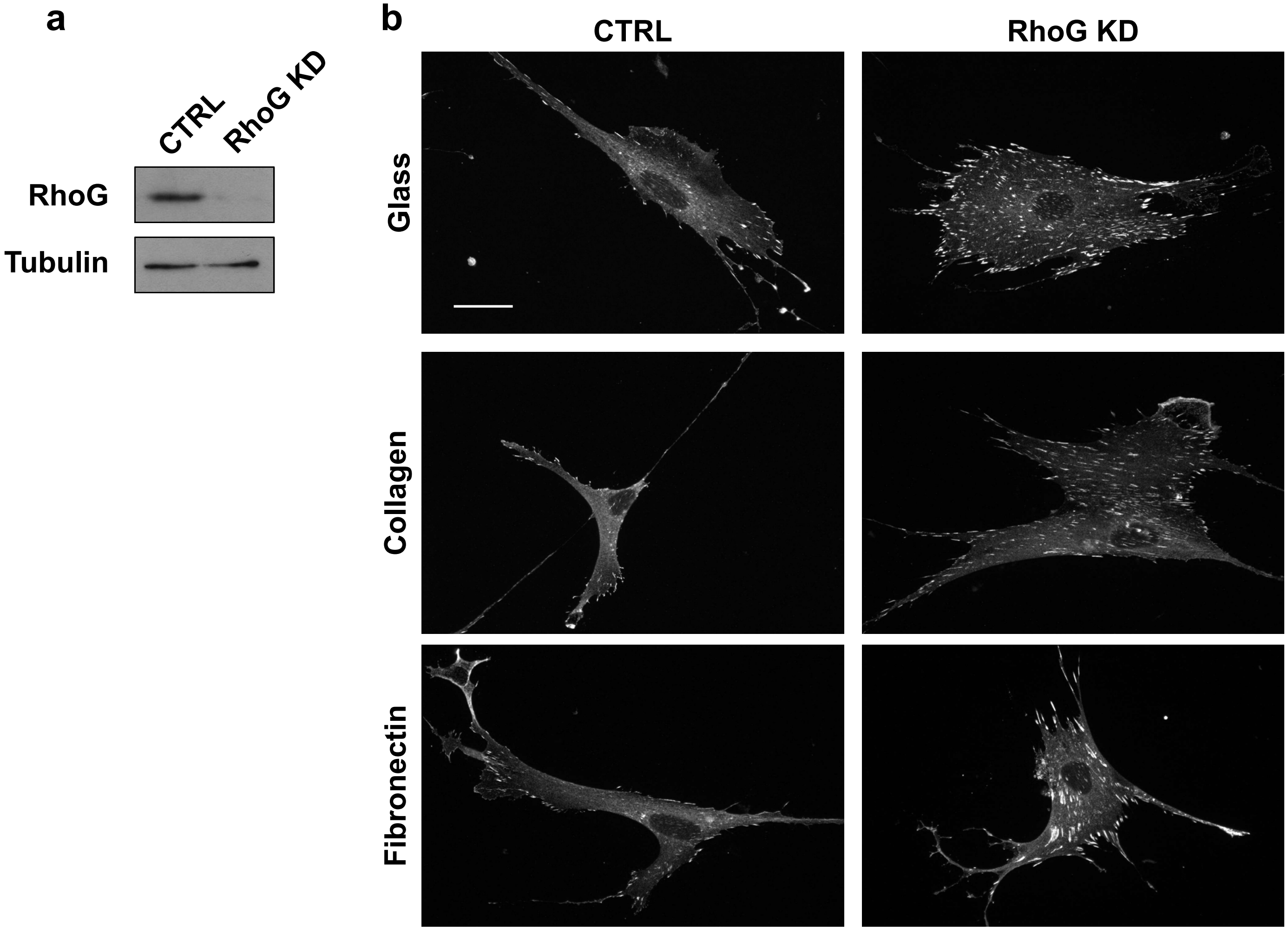

a
CTRL
RhoG KD
RhoG
Tubulin
b
CTRL
RhoG KD
Glass
Collagen
Fibronectin

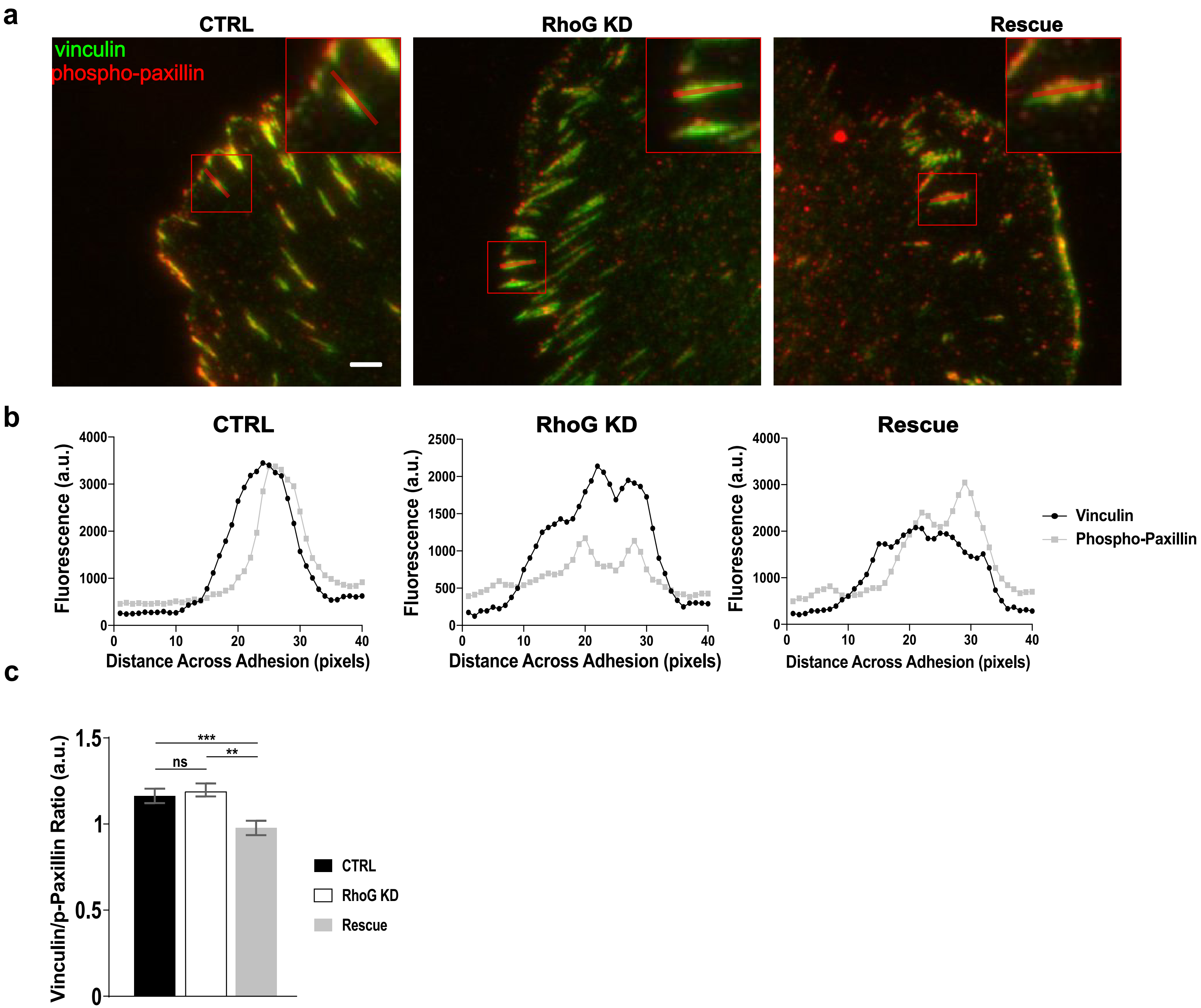

a
CTRL
RhoG KD
Rescue
vinculin
phospho-paxillin
b
CTRL
RhoG KD
Rescue
Fluorescence (a.u.)
Distance Across Adhesion (pixels)
Vinculin
Phospho-Paxillin
c
Vinculin/p-Paxillin Ratio (a.u.)
***
**
ns
CTRL
RhoG KD
Rescue

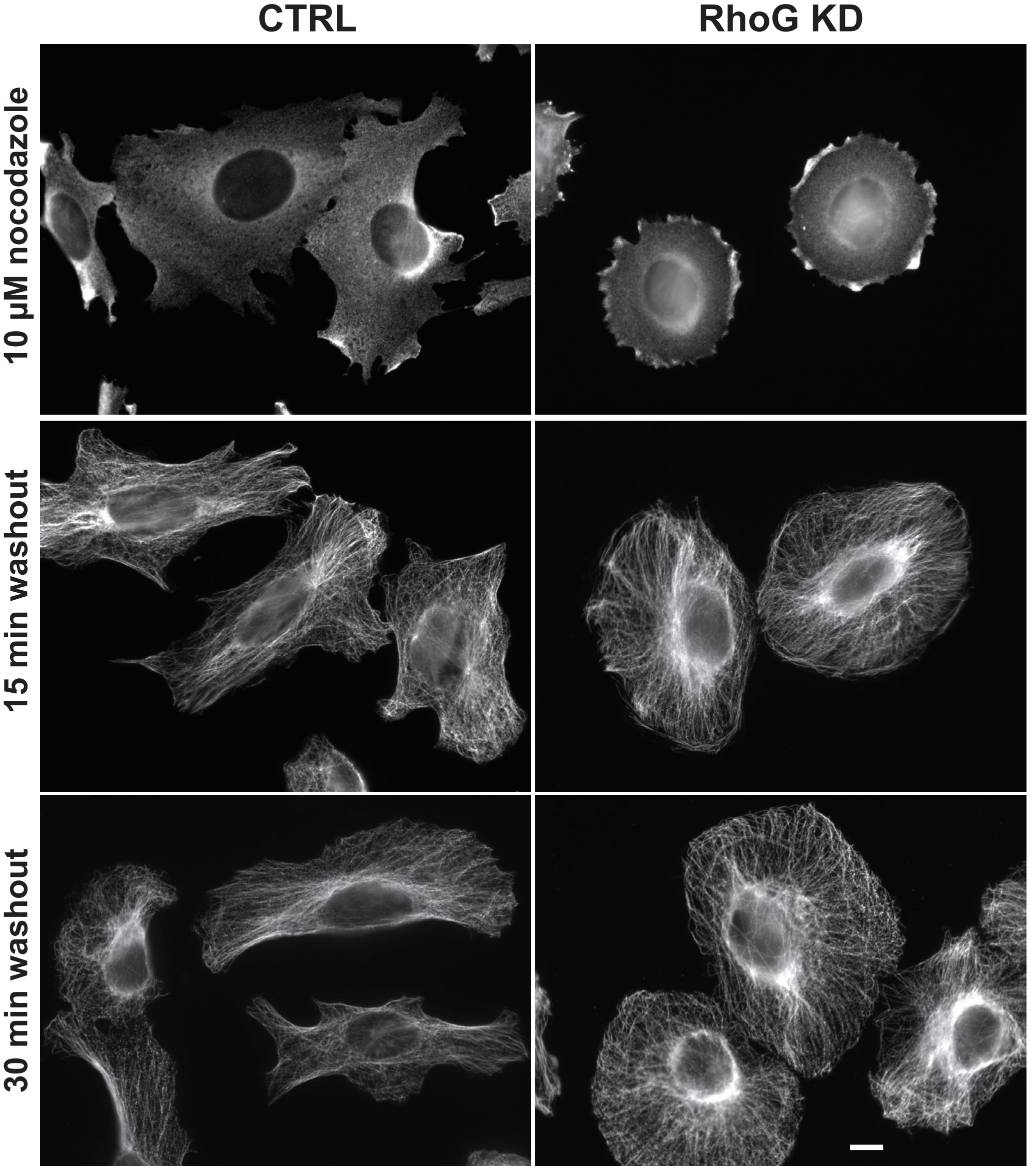

CTRL
RhoG KD
10 µM nocodazole
15 min washout
30 min washout
